# Construction and analysis of cotton (*Gossypium arboreum *L.) drought-related cDNA library

**DOI:** 10.1186/1756-0500-2-120

**Published:** 2009-07-02

**Authors:** Ling Zhang, Fu-Guang Li, Chuan-Liang Liu, Chao-Jun Zhang, Xue-Yan Zhang

**Affiliations:** 1 Key Laboratory of Cotton Genetic Improvement, Ministry of Agriculture, Cotton Research Institute, Chinese Academy of Agriculture Sciences, Anyang, Henan 455000, PR China

## Abstract

**Background:**

Drought is one of the most important environmental factors causing water stress for cotton, and it greatly limits cotton growth and crop productivity. So far only a few drought-tolerance genes have been functionally characterized in details, and most efforts on this topic have been made in model organisms. Therefore, to identify more drought-related genes in cotton plays a crucial role in elucidating the underlying mechanisms of drought tolerance as well as utilizing bioengineering techniques to improve the tolerance in this organism.

**Findings:**

Here we constructed a subtractive drought-tolerance cDNA library using suppressive subtractive hybridization (SSH). Through differential screening and bioinformatics analysis, we identified 392 positive clones with differential expression, corresponding 265 unique genes. By BLAST search against Genbank, we found that more than half of these EST sequences were homologous to those previously known drought-related genes and that there were 57 sequences with unknown functions, suggesting that many more genes are involved in this complex trait. Moreover, using RT-PCR, we examined the expression of nine representative candidate genes and confirmed that their expression levels were increased at different levels under drought stress.

**Conclusion:**

Our results show that drought tolerance is a complex trait in cotton, which involves the coordination of many genes and multiple metabolism pathways. The candidate EST sequences we identified here would facilitate further functional studies of drought-related genes and provide important insights into the molecular mechanisms of drought-stress tolerance and genetic breeding in cotton.

## Background

Drought stress is a crucial limiting factor for cotton production. Hence, enhancing drought tolerance has been one of the key issues in the practice of cotton planting. Breeding has been used to improve the drought tolerance of cotton, but so far the progress with this approach has been slow and limited [1]. Genetic engineering is another approach that could be used. However, with this approach, information about genes involved in cotton drought stress is required in advance. For this purpose, up-regulating key genes under drought stress may enhance drought tolerance.

Many drought-related genes have been reported in other plants, including maize, rice, and Arabidopsis. These genes can be mainly classified into two groups. One group contains proteins whose function is directly involved in stress tolerance, such as the enzymes required for photosynthesis enzymes [2,3], LEA proteins [4], mRNA binding proteins, protein enzymes, proline-rich proteins [5] and various proteases [6]. Proteins encoded by the other group appear to play regulatory roles, such as transcription factor MYB [7], zinc finger proteins [8,9], heat shock proteins (HSP) [10,11] and so on. Importantly, the wide range of these drought-stressed genes suggests that the responses to drought stress are rather complicated in plants.

Several techniques can be used to identify the genes expressed in response to drought stress, including DDRT-PCR [12], cDNA-AFLP [13], and suppression subtractive hybridization (SSH) [14]. Among these techniques, SSH appears to produce fewer false positives [15]. Therefore, we used SSH to construct a subtractive cDNA library of drought-stressed cotton.

## Results

### Construction of suppression-subtracted cDNA library

**Figure 1:**
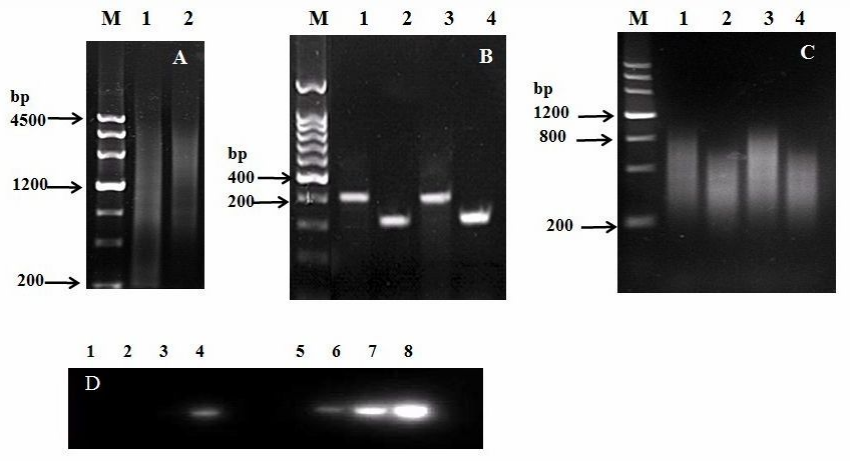
**The analysis on the SSH library**. Analysis of ds cDNA synthesis products and *Rsa *I digestion. Cotton double-stranded cDNA before (Lane 2) and after (Lane 1) *Rsa *I digestion. Lane M: DNA size markers. B) Analysis of ligation efficiency. Lane 1: PCR products using Tester 1-1 (Adaptor 1-ligated) as the template and the Histone 3 3' primer and PCR primer 1. Lane 2: PCR products using Tester 1-1 (Adaptor 1-ligated) as the template, and the Histone 3 3' and 5' primers. Lane 3: PCR products using Tester 1–2 (Adaptor 2R-ligated) as the template, and the Histone 3 3' primer and PCR primer 1. Lane 4: PCR products using Tester 1–2 (Adaptor 2R-ligated) as the template, and the Histone 3 3' and 5' primers. Lane M: DNA size markers. C) Results of PCR-select cDNA subtraction analysis. Lane 1: Forward secondary PCR products of unsubtracted. Lane 2: Forward secondary PCR products of subtracted. Lane 3: Reverse secondary PCR products of unsubtracted. Lane 4: Reverse secondary PCR products of subtracted. Lane M: DNA size markers. D) Analysis of subtraction efficiency using PCR. The subtracted and unsubtracted pools of cDNA were amplified by using primers for the constitutively expressed Histone 3 gene. PCR was performed on the subtracted (Lanes 1–4) or unsubtracted (Lanes 5–8) secondary PCR product with the Histone 3 5' and 3' primers. Lanes 1 & 5: 18 cycles; Lanes 2 & 6: 23 cycles; Lanes 3 & 7: 28 cycles; Lanes 4 & 8: 33 cycles.

Tester and driver cDNAs were reversely transcribed from the mRNA of the two sample pools, and the yield of double-stranded cDNA depended on the RNA quality. Fig. 1A shows the analyses of cDNA synthesis efficiency and *Rsa*I digestion. We then performed the PCR experiment to verify that at least 25% of the cDNAs had adaptors on both ends (Fig. 1B). This experiment was designed to amplify the fragments spanning the adaptor/cDNA junctions of Tester 1–1 and 1–2 with two gene-specific primers (Histone 3 3' and 5' primers) and PCR primer 1 according to the user's manual. If the band intensity of PCR products with one gene-specific primer and PCR primer 1 differed from that with two gene-specific primers by more than 4-fold, the ligation was less than 25% complete and will significantly reduce subtraction efficiency. After the secondary PCR analysis, the patterns of secondary PCR products from subtracted cDNA were denser than those of unsubtracted cDNA (Fig. 1C). We evaluated the subtraction efficiency by amplifying a housekeeping gene, Histone 3. By comparing the numbers of PCR cycles required for an equal amplification of the corresponding PCR products in the subtracted and unsubtracted cDNA samples, we demonstrated that differentially expressed genes were enriched in the subtracted libraries. In our experiment, 33 cycles were required to detect subtracted cDNA; whereas only 23 cycles were required for unsubtracted cDNA (Fig. 1D).

**Figure 2:**
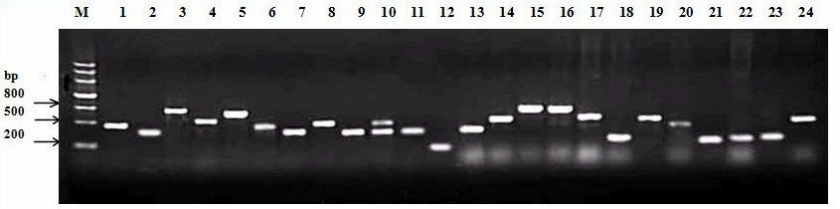
**The PCR analysis of partial clones in the subtracted library**. Lane 1–24: PCR products from different clones. Lane M: DNA size markers.

The PCR products of SSH were cloned into the pMD18-T vector and transformed into DH5α cells. The blue-white-spot screening showed that approximately 95% of the transformants contained the inserts. In total, 960 clones were obtained, and the subsequent colony PCR showed that the size of these inserts ranged from 200 to 800 bp (Fig. 2). Thus, we successfully constructed a putative drought-stress specific subtracted cDNA library of cotton seedlings.

### Differential screening of SSH library and analysis of SSH cDNA sequences

To eliminate the false positive clones and quantify the relative expression level of the cloned cDNAs more accurately, we performed further cDNA differential screening. The cDNA clones of differentially expressed genes were identified by successive screenings with the subtracted tester and driver as probes, respectively. Finally, we identified 392 positive clones whose tester expression level was significantly higher (> 3.0-fold) than that with driver.

### Bioinformatics sequence analysis

We sequenced 392 differentially expressed clones from different screening. The EST cluster analysis indicated that these sequences represented 265 unique ESTs. All the unique ESTs were submitted to the EST database of GenBank . These 265 unique ESTs included 41 contigs and 224 singletons. Based on homology search of BLASTX and BLASTN, among the 265 non-redundant sequences, 208 clones (78.5%) are homologous to known genes; and 57 clones (21.5%) are homologous to genes with unknown function or without matches in the database.

**Figure 3:**
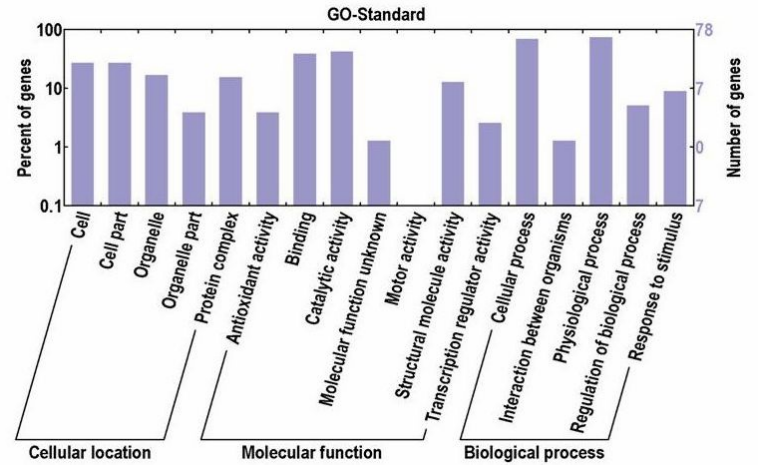
**The GO classification of cotton drought-tolerance genes**. The bar charts show the distribution of ESTs among three principal GO categories: cellular location, molecular function and biological process.

Based on GO annotation, 78 EST sequences were divided in to three organizing principal GO categories: cellular location, molecular function and biological process. Some ESTs were annotated with the three categories simultaneously. A gene product might be associated with or located in one or more cellular components; it is active in one or more biological processes, during which it performs one or more molecular functions [see Additional file 2]. As shown in Additional file 1 and Fig. 3, under the category of cellular location, the groups with the highest EST number are cell and cellar (21, 26.92%), respectively; under the category of molecular function, the highest EST number is 33 in catalytic activity (42.31%), followed by 30 in binding (38.46%); and under the category of biological process, cellular process (55, 69.24%) and physiological process (58, 74.36%) account for the majority of the annotated sequences. Meanwhile, no sequences were annotated with motor activity. The GO analysis suggested that the drought-related responses in cotton were mainly related to genes in metabolism and cellar structure.

**Figure 4:**
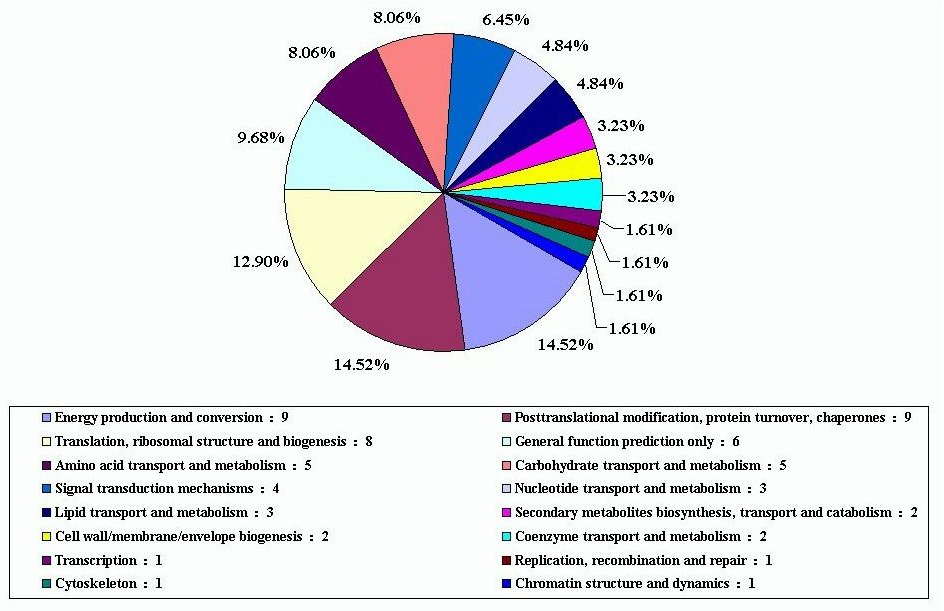
**The COG categories of drought-tolerance gene in cotton**. The drought-related gene sets differ in their distribution of COG categories (E-value < 1e-05). The pie chart is color-coded as per COG colors with the COG functional annotation, and represented the percentages of genes per COG category.

We also analyzed protein homology of 265 ESTs in the cluster of orthologous group (COG) database. Only 62 ESTs were found to have significant protein homologs (E-value < 1e-05) and were sorted into 16 groups according to the functional categories of the database [see Additional file 3] and Fig. 4. The largest EST set (14.52%) were assigned to the energy production, conversion and post-translational modification, protein turnover, chaperones category; and the second largest group (12.90%) were the genes involved in translation, ribosomal structure and biogenesis.

### RT-PCR

**Figure 5:**
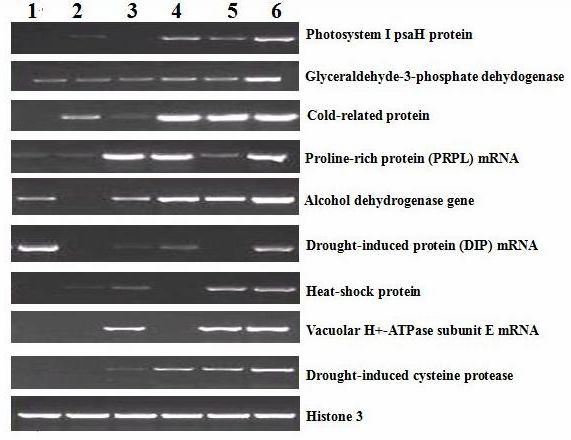
**The RT-PCR expression analysis of cotton nine clones isolated by SSH**. Gene-specific primers for nine clones were used to amplify a cDNA fragment of the corresponding gene after total RNA was reversely transcribed from drought-stressed cotton seedlings by 17% PEG6000. 1–6: The different time point from 1.5 h to 6.5 h. Histone 3 was used to normalize the amount of template in the PCR reactions.

We randomly selected nine representative ESTs that were previously reported to be associated with drought stress and evaluated whether these genes identified in SSH were differentially expressed in response to drought stress. Our RT-PCR results showed that most of these genes were indeed significantly up-regulated (Fig. 5).

## Discussion

In this study, we constructed a subtractive cDNA library containing water-stress induced transcripts in cotton seedlings. Based on our bioinformatics analysis, this SSH cDNA library contained many genes related to drought-stress tolerance. After differential screening, we identified 392 differentially expressed genes. With the homology search against Genbank, 265 of the genes had significant homologs in other plants, of which many are related to the drought-stress genes previously reported in Arabidopsis and maize.

The RT-PCR analysis on the EST expression showed that nine ESTs were up-regulated at different levels in drought-stress cotton seedlings. Among them, photosystem I psaH protein, and H+-ATPase-related gene [16] belong to the genes in cellular structure of leaves. These genes absorb and utilize water through adjusting the photosynthesis process. Under drought stress, the plant may reduce evaporation by closing the holes on the leaf surface, thereby fully utilizing water. Therefore, these two genes are highly induced.

The expression of glyceraldehyde-3-phosphate dehydogenase [17], alcohol dehydrogenase and drought-induced cysteine protease [18,19] were also obviously increased under drought stress. These three genes have been reported to be related to water stress in other plants. Our study confirms that these genes are also involved in the drought tolerance of cotton, suggesting that the response to drought is a very complex physiological and biochemical process and involves multiple metabolism pathways and many enzymes.

Moreover, some genes induced under drought stress were found to be associated with other environmental stresses [20,21], such as cold, salt, aluminium stress and so on. For example, our RT-PCR analysis confirmed one cold-related protein whose expression was slightly increased under drought stress. However, it should be noted that the number of such genes is very limited, which suggests that cotton is robust to drought stress and the damage is relatively moderate under drought stress.

Some studies show that under drought stress, the plant can improve the drought tolerance through adjusting osmoticum such as proline [22], trehalose [23] and glycinebetaine [24]. In our study, the mRNA expression of proline-related drought-induced protein (DIP) was significantly increased under drought stress, suggesting that this gene plays a key role in the drought-stress defense. Heat-shock proteins (e.g., HSP 90) are known to be important in protecting plants against stresses. They can bind to denatured proteins and maintain the soluble state, then facilitate to establish proper protein configuration and prevent unwanted protein from aggregating with the existence of Mg^2+ ^and ATP.

Compared with previously established SSH libraries in maize [25] and rice [26], our library contained many EST sequences annotated with drought tolerance. These ESTs appear to be involved in multiple metabolism pathways in the plant physiological and biochemical processes. In addition to known drought-induced genes, some differentially expressed genes are unknown, whose functional roles remain unclear and require further investigation in future.

## Conclusion

Our cDNA collection contains a broad repertoire of drought-related genes encoding proteins involved in both initial and physiological responses during normal and water stress stages of cotton seedlings. Our study would contribute to a better understanding the molecular mechanism of water-stress tolerance and facilitate the genetic manipulation of stress tolerance in cotton.

## Methods

### Plant material

Total RNA was obtained from the 3-to-6-leaf stage grown seedlings of cotton (*Gossypium arboreum *L.). Seeds were imbibed for one day in water at 30°C, and then sown in sterilized soil in plates for germination in Light-Emitted Feeding Box at 28°C. After 3–4 days, when appropriate, seedlings were transferred to soil and grown to the 3-to-6-leaf stage. In this study, 17% PEG6000 was used to induce "drought stress". Tissues were collected from seedlings maintained in water and in 17% PEG6000 at 1.5 hour after the start of the treatment and then hourly until 6.5 hour of the treatment. The tissues at different time points were pooled for RNA extraction.

### Extraction and purification of RNA

A modified CTAB method was used for RNA extraction. The absorption ratio of OD_260/280 _was used to verify the quality of RNA. For PCR-select DNA subtraction, mRNA was purified with the Oligotex™ mRNA Mini Kit (Qiagen, Germany).

### Construction of subtractive library

Suppression subtractive library was constructed according to the user manual of the PCR-Select™ cDNA Subtraction Kit (Clontech, USA). RNA from the seedlings maintained in water was used as the driver and RNA from the seedlings treated with 17% PEG 6000 was used as the tester. Double-stranded cDNA was prepared from mRNA. The cDNA was digested with *Rsa*I for about 3–4 h and then ligated to adapters 1 and 2R provided in the kit. Two rounds of hybridization and PCR amplification were processed to normalize and enrich differentially expressed cDNA. The subtractive products were inserted into the pMD18-T Vector (Takara, China) and transformed into DH5α cells. The positive white clones were then selected and cultured on LB containing 100 μg/ml ampicillin in 96-well plates at 37°C for 7 h. Then, 30% glycerol was added, and the culture was kept at -80°C.

### Amplification of cDNA inserts

The SSH products were amplified by PCR with nested primers provided in the kit. The reaction mixture contained 14.8 μl sterilized double distilled H_2_O (ddH_2_O), 2 μl 10 × ExTaq buffer (TaKaRa, China), 0.2 μl nested adapter 1, 2 R primers (10 μM each), 1.6 μl dNTP (0.5 mM), 0.2 μl ExTaq polymerase (TaKaRa, China), and 1 μl bacterial culture. PCR was performed as follows: 94°C for 5 min; 30 cycles at (95°C for 30 s; 68°C, 45 s; 72°C, 45 s; and 72°C, 5 min). The PCR products were electrophoresed on 1% agarose gel to confirm the amplification quality and quantity.

### Differential screening of subtracted cDNA library

After the subtracted cDNA library is obtained, it is important to confirm that individual clones indeed represent differentially expressed genes. For this purpose, differential screening of the subtracted library helps to eliminate false positives and to save time and effort. Such a screening was performed with the Clontech™ PCR-Select Differential Screening Kit (Clontech, USA). The cDNA inserts of the positive clones were amplified by PCR. The amplified products (1 μl) were then spotted on Hybond-N nylon membrane (Amersham, UK). After air drying, the membranes were denatured with 0.6 N NaOH, neutralized with 0.5 M Tris-HCl (pH 7.5), rinsed with sterilized water for 30 s, and then baked for 2 h. Sterilized water was used as the negative control, and a housekeeping gene, Histone 3 was used as the positive control. Probes were prepared with DIG High Prime DNA Labeling and Detection Starter Kit (Roche, Switzerland), and the following probes were used: 1) forward subtracted, 2) reverse subtracted, 3) unsubtracted tester, and 4) unsubtracted driver. We selected the fragments that only hybridized with the labelled tester cDNA or with at least three-fold higher signals on these membranes compared with those hybridized with the labelled driver cDNA for further sequencing.

### Bioinformatics sequence analysis

The positive clones identified by differential screening were sequenced at Beijing Genomics Institute. All the sequences were searched against the NCBI database with BLASTN and BLASTX . Functional classification of the ESTs was performed with the GO  and COG  tools.

### RT-PCR

Some cDNAs were selected for RT-PCR to confirm the results of differential screening. The housekeeping gene, Histone 3, was used as an internal standard. Primers were designed with the Primer 5.0 software. PCR was conducted as follows: 95°C, 5 min; 30 cycles (94°C, 30 s; 54°C, 1 min; and 72°C, 1 min); 72°C, 10 min. To perform a quantitative analysis on RT-PCR bands, the Chemigenius2 Bio Imaging System (Syngene, USA) was used.

## Competing interests

The authors declare that they have no competing interests.

## Authors' contributions

LZ conceived the work, designed the method, performed the analyses and wrote the manuscript. FGL have made substantial contributions to the conception and design of this study. CLL, CJZ and XYZ participated in planning and supervising the study. All authors have read and approved this manuscript.

## Supplementary Material

Additional file 1 **The GO classification of sequences**. GO data mining for drought-stressed genes with GO at the appropriate level. Table shows the number and percent of the EST sequences with GO-Standard. Click here for file

Additional file 2 **The GO annotation of sequences**. Table shows 78 EST sequences with Genbank accession, GO ID and GO annotation. Click here for file

Additional file 3 **The COG annotation of sequences**. Table shows 62 EST sequences with Genbank accession, COG name and COG annotation. Click here for file
