# Supplementary material for: Construction and analysis of cotton (Gossypium arboreum L.) drought-related cDNA library
Source: BMC Res Notes. 2009 Jul 2;2:120. doi: 10.1186/1756-0500-2-120 (PMC2714314; doi:10.1186/1756-0500-2-120)
Supplement: Additional file 1 — The GO classification of sequences. GO data mining for drought-stressed genes with GO at the appropriate level. Table shows the number and percent of the EST sequences with GO-Standard. [file 1756-0500-2-120-S1.doc]

| **GenBank Accession** | **GO Number** | **Go Annotation** | **GenBank Accession** | **GO Number** | **Go Annotation** |
| --- | --- | --- | --- | --- | --- |
| FK817152 | GO:0008270  GO:0016491 | zinc ion binding  oxidoreductase activity | FG357309 | GO:0003824  GO:0008152 | catalytic activity  metabolism |
| FK817153 | GO:0016020  GO:0009765 | membrane  photosynthesis, light harvesting | FG357312 | GO:0003700  GO:0006355 | transcription factor activity  regulation of transcription, DNA-dependent |
| FK817157 | GO:0009058 | biosynthesis | FG548040 | GO:0016020 | membrane |
| FK817158 | GO:0016616  GO:0006564 | oxidoreductase activity  L-serine biosynthesis | FG357313 | GO:0005489  GO:0006118 | electron transporter activity  electron transport |
| FK817141 | GO:0005488  GO:0006810 | binding  transport | FG548097 | GO:0009538  GO:0015979 | photosystem I reaction center  photosynthesis |
| FK817166 | GO:0004601  GO:0006979 | peroxidase activity  response to oxidative stress | FG548240 | GO:0004601  GO:0006979 | peroxidase activity  response to oxidative stres s |
| FK817171 | GO:0005489  GO:0005506  GO:0006118 | electron transporter activity  iron ion binding  electron transport | FG357315 | GO:0005634  GO:0016564  GO:0016481 | Nucleus  transcriptional repressor activity  negative regulation of transcription |
| FK817173 | GO:0046872 | metal ion binding | FG548034 | GO:0005525 | GTP binding |
| FK817175 | GO:0005515  GO:0008270 | protein binding  zinc ion binding | FK817181 | GO:0008289  GO:0006869 | lipid binding  lipid transport |
| FK817143 | GO:0008289  GO:0006869 | lipid binding  lipid transport | FG357300 | GO:0003677  GO:0006355 | DNA binding  regulation of transcription, DNA-dependent |
| FG357297 | GO:0005515  GO:0008270 | protein binding  zinc ion binding | FG547995 | GO:0004601  GO:0006979 | peroxidase activity  response to oxidative stress |
| FG357303 | GO:0000786  GO:0005634  GO:0003677  GO:0006334 | nucleosome  nucleus  DNA binding  nucleosome assembly | FG548004 | GO:0016020  GO:0004743  GO:0006096  GO:0009765 | Membrane  pyruvate kinase activity  glycolysis  photosynthesis, light harvesting |
| FG357307 | GO:0004365  GO:0051287  GO:0006096 | glyceraldehyde-3-phosphate dehydrogenase  NAD binding  glycolysis | FG548023 | GO:0004197  GO:0008234  GO:0006508 | cysteine-type endopeptidase activity  cysteine-type peptidase activity  proteolysis |
| FG548116 | GO:0005622  GO:0005840  GO:0003735  GO:0006412 | intracellular  ribosome  structural constituent of ribosome  protein biosynthesis | FG548118 | GO:0004497  GO:0005506  GO:0020037  GO:0006118 | monooxygenase activity  iron ion binding  heme binding  electron transport |
| **GenBank Accession** | **GO Number** | **Go Annotation** | **GenBank Accession** | **GO Number** | **Go Annotation** |
| FG548024 | GO:0005737  GO:0005554 | cytoplasm  molecular function unknown | FG548014 | GO:0005622  GO:0005840 | intracellular  ribosome |
| FG548029 | GO:0004672  GO:0005524  GO:0006468 | protein kinase activity  ATP binding  protein amino acid phosphorylation | FG548077 | GO:0004197  GO:0008234  GO:0006508 | cysteine-type endopeptidase activity  cysteine-type peptidase activity  proteolysis |
| FG548030 | GO:0006950 | response to stress | FG548075 | GO:0007165 | signal transduction |
| FG548039 | GO:0003824  GO:0006541 | catalytic activity  glutamine metabolism | FG548103 | GO:0008964  GO:0006099 | phosphoenolpyruvate carboxylase activity  tricarboxylic acid cycle |
| FG548043 | GO:0005622  GO:0005840  GO:0003735  GO:0006412 | intracellular  ribosome  structural constituent of ribosome  protein biosynthesis | FG548050 | GO:0005622  GO:0005840  GO:0003735  GO:0006412 | Intracellular  ribosome  structural constituent of ribosome  protein biosynthesis |
| FG548069 | GO:0016021 | integral to membrane | FG548084 | GO:0006464 | protein modification |
| FG548076 | GO:0005622  GO:0005840  GO:0003735  GO:0006412 | intracellular  ribosome  structural constituent of ribosome  protein biosynthesis | FG548098 | GO:0004568  GO:0006032  GO:0009613  GO:0016998 | chitinase activity  chitin catabolism  response to pest, pathogen or parasite  cell wall catabolism |
| FG548092 | GO:0004672  GO:0005524  GO:0006468 | protein kinase activity  ATP binding  protein amino acid phosphorylation | FG548105 | GO:0004672  GO:0005524  GO:0006468 | protein kinase activity  ATP binding  protein amino acid phosphorylation |
| FG548107 | GO:0006457 | protein folding | FG548115 | GO:0008299 | isoprenoid biosynthesis |
| FG548195 | GO:0004497  GO:0005506  GO:0020037  GO:0006118 | monooxygenase activity  iron ion binding  heme binding  electron transport | FG548269 | GO:0004672  GO:0004713  GO:0005524  GO:0006468 | protein kinase activity  protein-tyrosine kinase activity  ATP binding  protein amino acid phosphorylation |
| FG548119 | GO:0005622  GO:0005840  GO:0003735  GO:0006412 | intracellular  ribosome  structural constituent of ribosome  protein biosynthesis | FG548167 | GO:0005622  GO:0005840  GO:0003735  GO:0006412 | intracellular  ribosome  structural constituent of ribosome  protein biosynthesis |
| FG548137 | GO:0004637  GO:0009113 | phosphoribosylamine-glycine ligase activity  purine base biosynthesis | FG548139 | GO:0006904  GO:0016192 | vesicle docking during exocytosis  vesicle-mediated transport |
| FG548180 | GO:0016021  GO:0016192 | integral to membrane  vesicle-mediated transport | FG548192 | GO:0005634  GO:0003677 | nucleus  DNA binding |
| **GenBank Accession** | **GO Number** | **Go Annotation** | **GenBank Accession** | **GO Number** | **Go Annotation** |
| FG548146 | GO:0000015  GO:0004634  GO:0006096 | phosphopyruvate hydratase complex  phosphopyruvate hydratase activity  glycolysis | FG548152 | GO:0004672  GO:0005524  GO:0006468 | protein kinase activity  ATP binding  protein amino acid phosphorylation |
| FG548162 | GO:0006950 | response to stress | FG548138 | GO:0016787 | hydrolase activity |
| FG548163 | GO:0004672  GO:0005524  GO:0006468 | protein kinase activity  ATP binding  protein amino acid phosphorylation | FG548172 | GO:0004842  GO:0006464  GO:0006512 | ubiquitin-protein ligase activity  protein modification  ubiquitin cycle |
| FG548012 | GO:0004568  GO:0006032  GO:0009613  GO:0016998 | chitinase activity  chitin catabolism  response to pest, pathogen or parasite  cell wall catabolism | FG548041 | GO:0005622  GO:0005840  GO:0003735  GO:0006412 | intracellular  ribosome  structural constituent of ribosome  protein biosynthesis |
| FG548197 | GO:0004219  GO:0006508 | pyroglutamyl-peptidase I activity  proteolysis | FG548219 | GO:0016020  GO:0009765 | membrane  photosynthesis, light harvesting |
| FG548201 | GO:0009405 | pathogenesis | FG548208 | GO:0046872 | metal ion binding |
| FG548209 | GO:0005509 | calcium ion binding | FG548217 | GO:0005509 | calcium ion binding |
| FG548251 | GO:0016624  GO:0008152 | oxidoreductase activity, disulfide as acceptor  metabolism | FG548264 | GO:0016758  GO:0008152 | transferase activity, transferring hexosyl groups  metabolism |
| FG548268 | GO:0004194  GO:0006508  GO:0006629 | pepsin A activity  proteolysis  lipid metabolism | FG548262 | GO:0004842  GO:0006464  GO:0006512 | ubiquitin-protein ligase activity  protein modification  ubiquitin cycle |
| FG548209 | GO:0005874  GO:0043234  GO:0003924  GO:0005198  GO:0005525  GO:0007018  GO:0051258 | Microtubule  protein complex  GTPase activity  structural molecule activity  GTP binding  microtubule-based movement  protein polymerization | FG548145  FK817147  FG547994 | GO:0004550  GO:0005524  GO:0006183  GO:0006228  GO:0006241  GO:0046872  GO:0005198 | nucleoside diphosphate kinase activity  ATP binding  GTP biosynthesis  UTP biosynthesis  CTP biosynthesis  metal ion binding  structural molecule activity |
| FG548224 | GO:0005515  GO:0008270  GO:0006355 | protein binding  zinc ion binding  regulation of transcription, DNA-dependent | FG548271 | GO:0005509 | calcium ion binding |
| **Total gene number** | | | **78** | | |
