# Supplementary material for: Construction and analysis of cotton (Gossypium arboreum L.) drought-related cDNA library
Source: BMC Res Notes. 2009 Jul 2;2:120. doi: 10.1186/1756-0500-2-120 (PMC2714314; doi:10.1186/1756-0500-2-120)
Supplement: Additional file 2 — The GO annotation of sequences. Table shows 78 EST sequences with Genbank accession, GO ID and GO annotation. [file 1756-0500-2-120-S2.doc]

| GO | GO-Standard | | Number of gene | Percent of gene (%) |
| --- | --- | --- | --- | --- |
| Gene Ontology | Cellular location | Cell | 21 | 26.92 |
|  |  | Cell part | 21 | 26.92 |
|  |  | Organelle | 13 | 16.67 |
|  |  | Organelle part | 3 | 3.85 |
|  |  | Protein complex | 12 | 15.39 |
|  | Molecular function | Antioxidant activity | 3 | 3.85 |
|  |  | Binding | 30 | 38.46 |
|  |  | Catalytic activity | 33 | 42.31 |
|  |  | Molecular function unknown | 3 | 1.28 |
|  |  | Motor activity | 0 | 0 |
|  |  | Structural molecule activity | 10 | 12.82 |
|  |  | Transcription regulator activity | 2 | 2.56 |
|  | Biological process | Cellular process | 54 | 69.24 |
|  |  | Interaction between organisms | 1 | 1.28 |
|  |  | Physiological process | 58 | 74.36 |
|  |  | Regulation of biological process | 4 | 5.13 |
|  |  | Response to stimulus | 7 | 8.97 |
