# Supplementary material for: Construction and analysis of cotton (Gossypium arboreum L.) drought-related cDNA library
Source: BMC Res Notes. 2009 Jul 2;2:120. doi: 10.1186/1756-0500-2-120 (PMC2714314; doi:10.1186/1756-0500-2-120)
Supplement: Additional file 3 — The COG annotation of sequences. Table shows 62 EST sequences with Genbank accession, COG name and COG annotation. [file 1756-0500-2-120-S3.doc]

| **GenBank Accession** | **Portein name in COG** | **COG Annotation** |
| --- | --- | --- |
| **Energy production and conversion (9) METABOLISM** | | |
| FG548251 | slr1934 | Pyruvate/2-oxoglutarate dehydrogenase complex, dehydrogenase (E1) component, eukaryotic type, alpha subunit |
| FK817158 | SMa0478 | Lactate dehydrogenase and related dehydrogenases |
| FG548008 | YPO1502 | Zn-dependent alcohol dehydrogenases, class III |
| FK817152 | STM1627 | Zn-dependent alcohol dehydrogenases, class III |
| FK817164 | RC0729 | NifU homolog involved in Fe-S cluster formation |
| FG548103 | VC2646 | Phosphoenolpyruvate carboxylase |
| FG548020 | YOR332w | Archaeal/vacuolar-type H+-ATPase subunit E |
| FG548068 | CC3002 | Predicted oxidoreductases (related to aryl-alcohol dehydrogenases) |
| FG548155 | alr1526 | Ribulose bisphosphate carboxylase small subunit |
| **Posttranslational modification, protein turnover, chaperones (9) CELLULAR PROCESSES AND SIGNALING** | | |
| FG548262 | YGL087c | Ubiquitin-protein ligase |
| FG548107 | slr1251 | Peptidyl-prolyl cis-trans isomerase (rotamase) - cyclophilin family |
| FK817159 | YMR186w | Molecular chaperone, HSP90 family |
| FG548171 | YDR328c | SCF ubiquitin ligase, SKP1 component |
| FG548084 | SPAC6G10.11c_1 | Ubiquitin |
| FG357313 | YGR209c | Thiol-disulfide isomerase and thioredoxins |
| FG548124 | YBR082c | Ubiquitin-protein ligase |
| FG548148 | AF1946_2 | Cysteine protease |
| FG548176 | BH2830 | Glutathione peroxidase |
| **Translation, ribosomal structure and biogenesis (8) INFORMATION STORAGE AND PROCESSING** | | |
| FG548119 | YJL190c | Ribosomal protein S8 |
| FG548050 | SPAC8C9.08 | Ribosomal protein S7 |
| FG548034 | SPCP31B10.07 | Translation elongation factors (GTPases) |
| FG548043 | SPCC1183.08c | Ribosomal protein L1 |
| FG548014 | SPAC23A1.08c | Ribosomal protein L34E |
| FG548167 | SPAC664.05 | Ribosomal protein L13E |
| FG548116 | SPBC16C6.11 | Ribosomal protein L32E |
| FG548076 | YPL143w | Ribosomal protein L35AE/L33A |
| **General function prediction only (6) POORLY CHARACTERIZED** | | |
| FG548163 | SPAC23C11.11 | Serine/threonine protein kinase |
| FG548108 | BS_yfmJ | Putative NADP-dependent oxidoreductases |
| FG548087 | Cgl2652 | Uncharacterized NAD(FAD)-dependent dehydrogenases |
| FG548154 | CAC0404_1 | Serine/threonine protein kinase |
| FG548090 | PAB2118 | FOG: CBS domain |
| FG548029 | YDL101c | Serine/threonine protein kinase |
| **Amino acid transport and metabolism (5) METABOLISM** | | |
| FG357312 | VCA0765 | Threonine aldolase |
| FG357316 | sll1499_3 | Glutamate synthase domain 3 |
| FG547999 | cysE | Serine acetyltransferase |
| FG548011 | YLR355c | Ketol-acid reductoisomerase |
| FG357297 | cysE | Serine acetyltransferase |
| **Carbohydrate transport and metabolism (5) METABOLISM** | | |
| FG357307 | gapA | Glyceraldehyde-3-phosphate dehydrogenase/erythrose-4-phosphate dehydrogenase |
| FG548146 | SPBC1815.01 | Enolase |
| FK817161 | YPO2157 | Glyceraldehyde-3-phosphate dehydrogenase/erythrose-4-phosphate dehydrogenase |
| FG548004 | YAL038w | Pyruvate kinase |
| FG357314 | PA3467 | Permeases of the major facilitator superfamily |
| **Signal transduction mechanisms (4) CELLULAR PROCESSES AND SIGNALING** | | |
| FG548162 | Cgl2878 | Universal stress protein UspA and related nucleotide-binding proteins |
| FG548217 | SPAC3A12.14 | Ca2+-binding protein (EF-Hand superfamily) |
| FG548080 | MJ0531 | Universal stress protein UspA and related nucleotide-binding proteins |
| FG548030 | Cgl2878 | Universal stress protein UspA and related nucleotide-binding proteins |
| **Nucleotide transport and metabolism (3) METABOLISM** | | |
| FG548137 | SMc00993 | Phosphoribosylamine-glycine ligase |
| FG548138 | PA0441 | Dihydroorotase and related cyclic amidohydrolases |
| FG548145 | sll1852 | Nucleoside diphosphate kinase |
| **Lipid transport and metabolism (3) METABOLISM** | | |
| FK817176 | CC1723 | Enoyl-CoA hydratase/carnithine racemase |
| FK817140 | CC1723 | Enoyl-CoA hydratase/carnithine racemase |
| FG548200 | SMc00264 | Dehydrogenases with different specificities (related to short-chain alcohol dehydrogenases) |
| **Secondary metabolites biosynthesis, transport and catabolism (2) METABOLISM** | | |
| FG548195 | BH0579 | Cytochrome P450 |
| FG548118 | mlr5192 | Cytochrome P450 |
| **Cell wall/membrane/envelope biogenesis (2) CELLULAR PROCESSES AND SIGNALING** | | |
| FG548261 | BSgalE | UDP-glucose 4-epimerase |
| FK817157 | all4985 | Glycosyltransferase |
| **Coenzyme transport and metabolism (2) METABOLISM** | | |
| FG548115 | YJL167w | Geranylgeranyl pyrophosphate synthase |
| FG548091 | NMB1799 | S-adenosylmethionine synthetase |
| **Transcription (1) INFORMATION STORAGE AND PROCESSING** | | |
| FG548192 | ECU11g1690 | Myb superfamily proteins, including transcription factors and mRNA splicing factors |
| **Replication, recombination and repair (1) INFORMATION STORAGE AND PROCESSING** | | |
| FG548186 | PAE3199 | A/G-specific DNA glycosylase |
| **Cytoskeleton (1) CELLULAR PROCESSES AND SIGNALING** | | |
| FG548071 | SPBC26H8.07c | Tubulin |
| **Chromatin structure and dynamics (1) INFORMATION STORAGE AND PROCESSING** | | |
| FG357300 | SPAC57A10.09c | Chromatin-associated proteins containing the HMG domain |
| **Total gene number** | | **62** |
